# Development, validation and application of a 3D printed model depicting adenoid hypertrophy in comparison to a Nasoendoscopy

**DOI:** 10.1186/s13005-020-00216-4

**Published:** 2020-03-09

**Authors:** Claudine Thereza-Bussolaro, Manuel Lagravère, Camila Pacheco-Pereira, Carlos Flores-Mir

**Affiliations:** School of Dentistry, University of Alberta, 11405 87Ave NW, 5528 Edmonton Clinic Health Academy,, Edmonton, AB T6G1C9 Canada

**Keywords:** Cone-beam computed tomography, Printing, three-dimensional, Adenoids, Nasopharynx, Child, Adolescent

## Abstract

**Background:**

The exploration of tridimensional (3D) technology of computational tomography and the development of valid 3D printed models may improve the assessment of adenoid obstruction. The identification of an enlarged adenoid in childhood would streamline the referral of appropriately selected cases to an otolaryngologist, leading to early treatment of affected children when indicated. The objective of this study is to validate the use of a 3D printed model depicting adenoid hypertrophy based on the pediatric otolaryngologist, head and neck surgeon (OHNS) participants assessment.

**Methods:**

A cross-sectional study was performed to develop and validate 3D depictions, including print-outs, of the nasopharynx including different degrees of Adenoidal Hypertrophy (AH). The print-outs were obtained from 14 Cone-beam computed tomography (CBCT) scans of 14 children (12 boys, 2 girls; mean age of 10.61 years) representing grades 1, 2, 3, and 4 nasopharyngeal adenoidal obstructions, according to a previously Nasoendoscopy-graded (NE) classification by a licensed OHNS. The prevalence of AH in this study was 36%. Two OHNS were recruited to assess the print-outs representing two different representations of the nasopharyngeal airway, the lumen (LU) and adenoid mass (AD). LU and AD were visualized in 2D - pictures- and in 3D – printed prototypes. Intraclass correlation was used to assess intra- and inter-reliability. The validity of our depictions was analyzed through comparison (accuracy and correlation) to the reference standard (NE). The data were clustered to calculate the sensitivity (Se), specificity (Sp), positive predictive value (PPV), and negative predictive value (NPV). Cross-tab and Pearson’s T-test were performed.

**Results:**

Overall, the 3D depiction tools showed different diagnostic capabilities. AD representations showed strong (AD 2D, 75%) to almost perfect (AD 3D, 88%) accuracy compared to NE. Excellent sensitivity and specificity were observed for the AD 3D (100, 70%), as well as adequate PPV and NPV (66 and 97% respectively), with only 5% of false-negative cases.

**Conclusion:**

The use of Dolphin software for the acquisition of a 3D printed prototype of the nasopharyngeal adenoidal region seems promising. These prototypes may be a practical and readily available alternative for the assessment of the nasopharyngeal obstructed area. CBCT in children must be taken under strong solid indications. Early referral to an OHNS for a full assessment remains the main objective in children with unclear symptoms.

## Background

Signs of sleep-disordered breathing (SDB) are considered relatively common among children. SDB represents a myriad of related disorders ranging from snoring to upper airway resistance syndrome to an obstructive sleep apnea syndrome [1]. Poor school performance, gasping for breath at night, and snoring are some of the signs reported by caregivers. A narrow upper airway has been associated with pediatric OSAS, and one of the more likely cause is adenotonsillar hypertrophy. Adenoidectomy could be indicated when it is associated with nasopharyngeal obstruction [2]. Thus, identification of an enlarged adenoid in childhood would streamline the referral of appropriately selected cases to an otolaryngologist, head and neck surgeon (OHNS), leading to early treatment of affected children when indicated.

The usefulness of diagnostic tools and referral algorithms for the detection of enlarged adenoid and nasopharyngeal obstruction has been developed and investigated over the years [3]. The evaluation of nasopharyngeal obstruction is done either estimated subjectively by direct visualization or objectively by mean of direct measurement in pertinent imaging [3]. The antrum-adenoidal space, the ratio between adenoid, the nasopharyngeal space, and the measured choanal obstruction space are examples of objective measurements. Adenoid grading methods also vary from simply categorical “normal or enlarged” [4, 5], “small, moderate and large” [6] to ordinal scales in three [7] or four grades [8].

Flexible fiberoptic nasal endoscopy (NE) is an imaging method used for multiple purposes in a routine OHNS practice [9]. It has shown a sensitivity of 92% and specificity of 71% for adenoidal hypertrophy obstruction detection [10]. Cone-beam computed tomography (CBCT) is becoming part of the routine of orthodontic records and it has been explored for evaluating adenoid size [11–14] in comparison to NE. Therefore, Digital Images and Communication in Medicine (DICOM) files would be readily available for adenoid hypertrophy assessment in some cases when there is suspicion of upper airway (UAW) obstruction. The exploration of 3D print outs of the nasopharyngeal area has not been explored, it could streamline the diagnostic process, especially in cases where the patients have already a recent CBCT in which a hypertrophic adenoid is suggested. It would also substitute the need for an additional NE in selected cases and would be useful in remote regions where access to an OHNS specialist is absent.

## Material and methods

### Study design and protocol

**Fig. 1:**
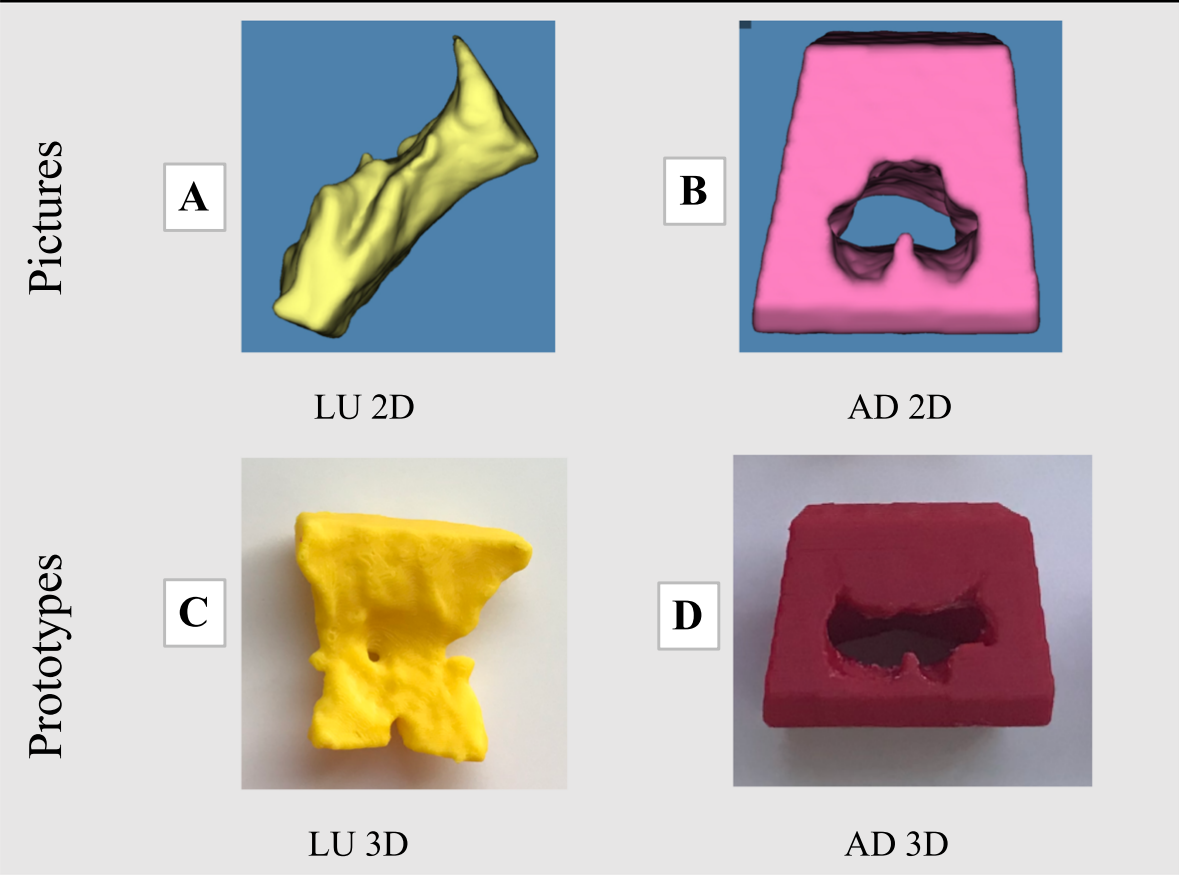
3D depictions

Four depictions (two 2D, and two 3D) of the nasopharyngeal adenoidal area were created per included participant from the sample of available cases (Fig. 1). The manufacturing techniques of the prototypes were described in Appendix A. A flowchart of the sampling reasoning and study design can be seen in Appendix B.

### Ethics

A prospective protocol for validation of the 3D printouts was proposed and ethical approval was obtained through the research ethics committee at the University of Alberta under protocol number Pro00082445.

### DICOM sampling

The selected sample consisted of CBCT scans of 14 children representing grades 1, 2, 3, and 4 nasopharyngeal obstructions, according to a previously NE-graded classification by a licensed OHNS [11]. A total of 12 boys and 2 girls with a mean age of 10.61 years (7.2–15.7 years old, SD = 2.99) were considered. The selected sample consisted of six cases of grade 1 (42.9%), three cases of grade 2 (21.4%), three cases of grade 3 (21.4%), and two cases of grade 4 (14.3%), based on the distribution of the Parikh grading system classification [8]. The prevalence of AH in the present study was 36%, which is very similar to the percentage prevalent in the pediatric population, 34.46% [15]. Converting the sample to a clinical classification as non-enlarged (Grades 1 and 2) and enlarged (grades 3 and 4), the sample ended up having 9 cases in the non-enlarged group with a mean age of 10.22 years (SD 3.25) and 5 cases in the enlarged group with a mean age of 11.31 (SD 2.63). Appendix A of this research project shows the demographics and descriptive statistics of the selected sample. Appendix B contains a flowchart of sample selection, inclusion, exclusion criteria, and eligibility.

### OHNS sampling

A sample of two evaluators was recruited at the Department of Otolaryngology-Head and Neck Surgery of the Faculty of Medicine and Dentistry of the University of Alberta, In Canada. The participants had to be registered OHNS specialists in the province of Alberta. All were sent a letter of invitation by email, it was made clear through informed consent that the study was voluntary, and the material from the data collection was anonymized.

### Reference standard

Our reference standard method was based on previously performed NE exams and details are described in a previous study [11].

### Procedure

**Fig. 2:**
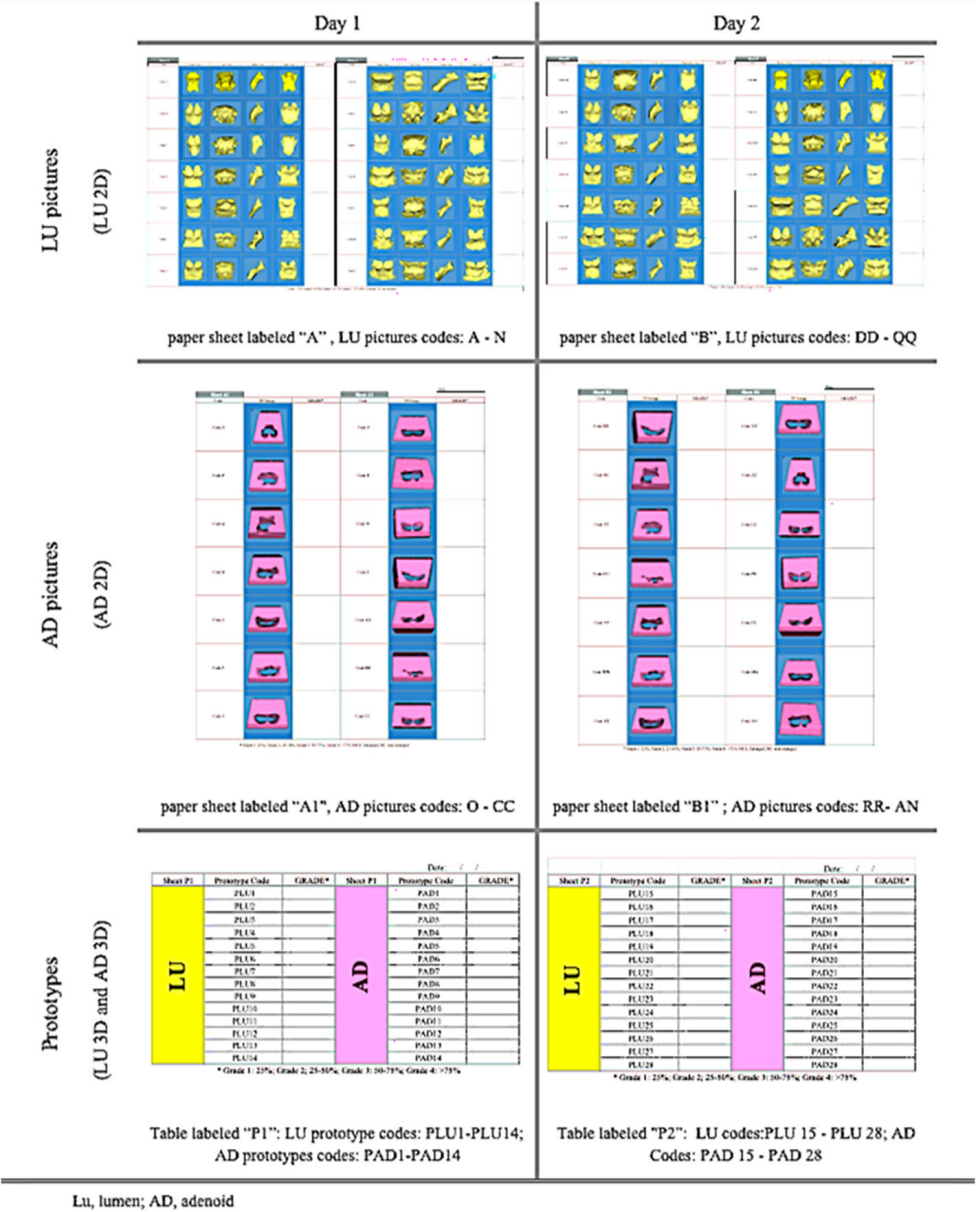
Assessment of the 3D depictions

The 3D depictions of the pharyngeal adenoidal obstruction included two different anatomic regions of the nasopharyngeal airway, the lumen (LU) and adenoid mass (AD). LU and AD were visualized in 2D - pictures- and in 3D - prototypes. One member of the research team (CTB) took the 3D depictions alongside with a guidance sheet and with a cheat sheet containing the grading system - to one participant at a time. Each participant was assessed two times with an interval of 1 week between the assessments, The 3D prototypes were coded in a way that the same prototype received two different codes depending on the day it was assessed, as shown in Fig. 2 The participants were given different sheets and codes, according to the day of assessment, and they graded the level of obstruction of the nasopharynx, accordingly to four grades of AH through NP assessment using the Parikh et al., 2006 grading system [8].

### Statistical analysis

Statistical analyses were done using the Statistical Package for the Social Science (IBM, version 25; SPSS Inc., Armonk, NY, USA). Microsoft® Excel for Mac, version 15.27 was used to obtain any necessary averages and graphs. The intraclass correlation coefficient (ICC) was used to assess the intra and inter-reliability between the two evaluators. We followed the interpretation of poor agreement = 0–0.2; fair agreement = 0.3–0.4; moderate agreement = 0.5–0.6; strong agreement = 0.7–0.8; almost perfect agreement = > 0.8 [16]. The validity of our depictions was analyzed through comparison (accuracy and correlation) between our tools results and the reference standard - NE. To calculate the sensitivity, specificity, positive predictive value (PPV), and negative predictive value (NPV), we clustered the results in non-enlarged and enlarged. Cross-tab and Pearson’s c-test were performed. MANOVA was performed to find the power description and effect size (partial eta-squared - **η**_**p**_^**2**^) of the study. The level of significance and confidence interval (CI) was set at 0.05.

## Results

One hundred and twelve (*n* = 112) adenoidal nasopharyngeal assessments were evaluated by each OHNS participating in this study. A total of 28 OHNS were invited and two agreed to participate. The participants evaluated the adenoid size of 14 patients represented in 4 different ways: LU 2D, AD 2D, LU 3D, AD 3D as shown in Fig. 1 respectively A, B, C, D. Overall, an almost perfect overall agreement was observed for the 112 possible agreements in adenoid grading from the two examiners scoring in grading system, inter-rater reliability ICC mean = 0.88 (95% CI, 0.76–0.95), and in the clinical classification of enlarged and non-enlarged, ICC mean = 0.87 (95% CI, 0.75–0.95). The lower bound of the agreement still implied the “strong agreement” grading.

### Reliability and statistical power

Statistical power analysis of the evaluations based on the grading system [8], was high for each shared visualization tool. Based only on enlarged and non-enlarged classification, it was also high for all 3D depictions (> 0.92); however, 0.84 for LU 3D which is still a high power. The effect size was large for all 3D depictions in the grading classification, although for the clinical classification effect size was large for AD 3D, medium for AD 2D; however, it was small for LU 2D and LU 3D, and as seen in Table 1. The degree of consistency and agreement, verified through intra and inter reliability, in with OHNS evaluated the depictions were observed through intra-rater reliability by grading score and by clinical classification as below as shown in Table 1.

**Table 1:** OHNS Reliability

	Grading System^a^				Clinical Classification^b^			
	OHNS 1		OHNS 2		OHNS 1		OHNS 2	
3D depictions	INTRA	*P*-value (95%)	INTRA	*P*-value (95%)	INTRA	*P*-value (95%)	INTRA	*P*-value (95%)
LU 2D	ICC = 0.00; CI: 0 – 0.55	NS	ICC = 0.88; CI: 0.63 – 0.96	*P* < 0.001	ICC = 0.92; CI: 0.78 – 0.98	*P* < 0.001	ICC = 0.93; CI: 0.78 – 0.98	*P* < 0.001
AD 2D	ICC = 0.97; CI: 0.92 – 0.99	*P* < 0.001	ICC = 0.93; CI: 0.79 – 0.98	*P* < 0.001	ICC = 0.93; CI: 0.78 – 0.98	*P* < 0.001	ICC = 0.93; CI: 0.78 – 0.98	*P* < 0.001
LU 3D	ICC = 0.86; CI:0.57–0.95	*P* < 0.0051	ICC = 0.64; CI: 0.0 – 0.88	*P* < 0.001	ICC = 0.93; CI: 0.78 – 0.98	*P* < 0.001	ICC = 0.73; CI: 0.19 – 0.913	*P* < 0.001
AD 3D	ICC=0.84; CI:0.52 – 0.95	*P* < 0.001	ICC = 0.71; CI: 0.12 – 0.91	*P* < 0.001	ICC = 0.75; CI: 0.23 – 0.91	*P* < 0.001	ICC = 0.16; CI: 0.0 – 0.73	NS
	Grading System^a^				Clinical Classification^b^			
	OHNS 1 & OHNS 2							
3D depictions	INTER	*P*-value (95%)	Observed Power	Effect size	INTER	*P*-value (95%)	Observed Power	Effect size
LU 2D	ICC=0.64, CI: 0 - 0.68	NS	1	96%	ICC=0.26, CI: 0.0 - 0.74	NS	0.92	53%
AD 2D	ICC=0.88, CI: 0.64 - 0.96	*P* < 0.001	1	95%	ICC=0.93, CI:0.78 - 0.98	*P* < 0.001	0.98	62%
LU 3D	ICC=0.59, CI: 0 - 0.87	*P* < 0.005	1	95%	ICC=0.26, CI:0.0 - 0.77	NS	0.84	47%
AD 3D	ICC=0.79, CI:0.37 - 0.93	*P* < 0.001	1	97%	ICC=0.73, CI: 0.14 - 0.91	*P* < 0.005	1	75%

*OHNS* otolaryngologist; ^a^accordingly to Parikh et al, 2006 (grade 1, 2 3 and 4); ^b^enlarged and non-enlarged

### Accuracy test

In summary, by the grading system according to Parikh [8], moderate but not statistically significant accuracy was found for LU 2D, a statistically significant and strong accuracy was observed for AD 2D, a moderate but not significant accuracy was observed for LU 3D, and a statistically significant and almost perfect accuracy was found for AD 3D. Accuracy by the clinical classification of enlarged and non-enlarged was poor and not statically significant for LU 2D, statistically significant and moderate for AD 2D, also moderate but not statically significant for LU 3D, and statistically significant and almost perfect for AD 3D. Therefore, both 2D depictions (LU 2D and AD 2D) showed a decrease in accuracy under clinical classification vs grading system Table 2 shows the accuracy and correlation results.

**Table 2:**
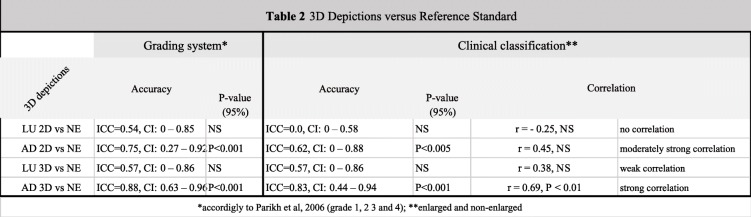
3D depictions versus reference standard

### Sensitivity, specificity, positive and negative predictive values

Low sensitivity and high specificity were found for LU 2D, high sensitivity and high specificity was found for both AD 2D and AD 3D, and low sensitivity and low specificity for LU 3D. In a sample with an AH prevalence of 36%, with a positive test for enlarged adenoid the chances of a patient who actually have an enlarged adenoid to be tested positive increases from 36 to 54% in the AD 2D, to 47% in the LU 2D, and to 66% in the AD 3D. Nevertheless, the chances of a patient who has an enlarged adenoid to test positive for LU 2D decrease from 36 to 29%. Regarding NPV results, the chances of the patient with a negative test for enlarged adenoid who does not have enlarged adenoid increases from 64 to 85% for AD 2D, to 72% for LU 3D, and 97% for AD 3D. Nevertheless, the chances of a non-enlarged patient to be tested as “non-enlarged” decreases from 64 to 59% in LU 2D. Table 3 shows a summary of the diagnostic capabilities results.

**Table 3:**
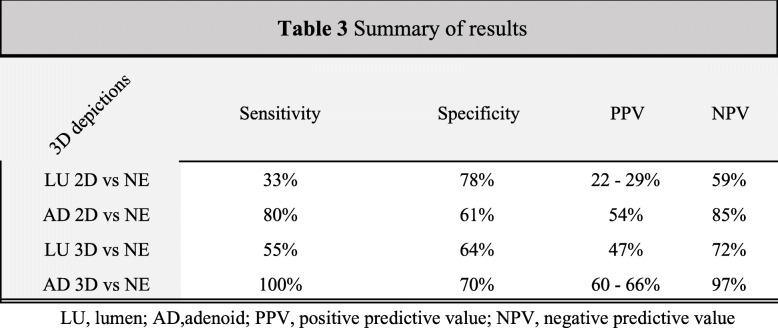
Summary of Results

## Discussion

The need for early referral, diagnosis and management of AH in children has been suggested in the literature over the years. Dentists should consider the possibility of referring the patient for a full OHNS assessment if any potential nasopharyngeal obstruction is identified. Direct clinical visualization of some pharyngeal areas can be limited, alternative nasopharyngeal image approaches could be used to improve the screening for potential obstruction. The exploration of 3D technology of computational tomography, and the development of valid 3D printed models may improve the assessment of adenoid obstruction. Regarding individual visualization tool’s performance – LU 2D, AD 2D, LU 3D, and AD 3D - both the picture (AD 2D) and the prototype (AD 3D) representing the adenoid and soft tissue were in general terms reliable (ICC > 0.75) and accurate while comparing with our reference standard, ICC > 0.80. This is probably because the examiner can subjectively calculate the lumen space comparing it to adjacent anatomic structures. Besides, the “AD assessment” type of view is similar to the view OHNS has on the NE exam; thus, the examiners were more familiar with this view.

Various methods have been developed over the years to assess adenoid sizes [9, 17], mostly based on the available space (lumen) in the nasopharynx around the adenoids, and not specific on the real size of the adenoid tissue. The exploration of 3D printing has not been stressed, our methodology evaluated the performance of two different depictions, lumen and adenoid tissue. And the late, showed better performance, probably because it allowed a view of the relationship between the adenoid and the nasopharynx space available.

In comparison with the NE assessment, AD 3D and AD 2D showed better statistical results in both grading systems and clinical classifications; furthermore, AD 3D presented almost perfect agreement in both of them. Additionally, AD 2D and AD 3D visualization tools showed respectively a moderately and strong correlation with our reference standard. Therefore, both AD 2D and AD 3D visualization tools allowed for an accurate grading of the adenoidal nasopharyngeal area. We assume that is probably due to the similarity of this view with the NE view, in which they are habituated to.

Between the two depictions of the adenoid tissue and soft tissue - AD picture and prototype - the later presented slightly better results for accuracy, correlation, specificity, and sensitivity. We hypothesize that this could be due to the 3D characteristic of the prototypes which allowed touching and looking at the real depth of the nasopharyngeal and the adenoid along with its relationship with adjacent structures. Although, while the prototype showed better performance, in a clinical setting the access to 3D printers is limited, therefore the application of AD 2D by health professionals would be more realistic and would per se help streamline the affected patients to the care of a specialist.

The diagnostic capability of the assessed visualization tools as a diagnostic test for AH was statistically calculated. Sensitivity is the ability of a test to identify the adenoid enlarged cases, while specificity is the test’s ability to identify all non-enlarged cases. Excellent sensitivity and specificity were observed for the AD 3D (100, 70%). A sensitivity of 100% means that our tool was able to identify all the cases with AH and that the number of false negatives was low. Major et al., 2014 [17] stated that lower sensitivity is acceptable for AH, at the same time the authors contradict themselves stating that “low rate of false-negative cases” is preferred to not miss the undiagnosed patients. We believe the authors meant a higher sensitivity is preferred not a low sensitivity, because it leads to a low rate of false-negative cases. After all, a link between AH and upper airway obstruction has been reported [18, 19], such obstruction may lead to SBD, to a compromised quality of life and general physical conditions [9, 20], and that a delay in treatment also increases the need for more complex medical interventions [21, 22]. Therefore, the AD 3D depiction seems to have achieved the study goal for correctly identifying enlarged adenoids, and also beat a previously [17] set up cutoff values for specificity at 90%.

The accuracy of adenoid tests has been investigated in a systematic review [17]. The author found a great variability between diagnostic tools compared to NE, for specificity ranging from 34 to 97%, and for sensitivity, from 22 to 100%. The best results were seen in a videofluoroscopy study [23] -100% sensitivity and 93% specificity, and MDCT study- 92% for sensitivity and 97% for specificity. However, they carry the disadvantage of higher radiation compared to CBCT. On the other hand, the clinical examination does not expose the patient to ionizing radiation; however, it showed a poor sensitivity of 22% and an excellent specificity of 88% [17]. Thus, since a CBCT is not an independent exam, meaning, it would always be complemented by a specialist consultation and clinical examination, it would definitely improve the low sensitivity of the clinical examination.

In a population-based setting with a prevalence of 35.7%, the probability of patients who truly have enlarged adenoid (PPV) to be identified by the 3D depictions was higher for the AD 3D with a PPV between 60 and 66%; AD 2D showed a good probability (PPV = 54%) as well. The probability of patients who truly do not have enlarged adenoid (NPV) to be identified by our tools was 97% for AD 3D, and 85% for AD 2D, and 72% for LU 3D. The worst performance was observed for the picture of the lumen (LU 2D) PPV = 22–29% and NPV = 59%, since their probability was below the prevalence of enlarged (36%) and non-enlarged (64%) in the population.

The awareness of the 2D and 3D depictions’ screening capability associated with the support of the OHNS community can lead to a prioritization of the assistance to likely affected patients. Besides, in a scenario where a patient already has a CBCT of the craniofacial structures taken for another reason, OHNS can rely on the CBCT 3D depiction which might eliminate the need for an NE in some cases, for instance, non-surgical cases. Therefore, multidisciplinary cooperation can fast-track referral to and consequently the management of affected individuals by an OHNS. Altogether those actions can benefit individual and overall community health, since wait times for specialist consultation and treatment delay may increase the rate of deterioration in general physical conditions, and also can lead to more complex medical interventions [21].

Finally, it should be stressed out that the prescription of CBCT imaging for children must be based on strong indications and a restrict selection criteria. Children with unclear symptoms should be referred to a specialist who will decide about the preferred diagnostic method.

### Limitations

The main limitation was the sample size. We contacted 28 OHNS and residents; only 6 answered the emails, and among them, 4 declined to participate due to scheduling problems, and 2 accepted to participate. Another limitation was the reference standard that was used. The models were selected based on the retrospective grading of only one evaluation, in which neither inter-rater reliability (agreement) nor intra-rater reliability (consistency) was assessed.

## Conclusion

Our findings support the validation of the use of 3D printed model depictions of the adenoid obstruction of the nasopharynx. Accuracy was found in two 3D printed models’ depictions-LU 3D and AD 3D- and in one 3D picture depiction-AD 2D. Screening capabilities of the four 3D depictions tools are presented below:

- LU 2D visualization tool is reliable between repeated evaluations and has high specificity; however, it is not accurate, has low sensitivity, and has poor performance on PPV and NPV;
- AD 2D visualization tool is reliable between repeated evaluations, and accurate compared with NE (reference standard). It also has high sensitivity and specificity;
- LU 3D visualization tool is reliable between repeated evaluations and showed moderate accuracy, low sensitivity and specificity;
- AD 3D visualization tool is reliable and accurate for evaluating AH compared with NE (reference standard). This depiction presented the highest sensitivity, and the highest values for PPV and NPV compared to the other visualization tools.

## Supplementary information

**Additional file 1.** Development of a 3D printed model of the nasopharyngeal adenoidal area using CBCT (Methodology).

**Additional file 2.** Flowchart of the sampling reasoning and study design.

## Data Availability

The datasets used and/or analyzed during the current study are available from the corresponding author on reasonable request.

## Abbreviations

2D: Two-dimensional
3D: Three-dimensional
AD: Adenoid mass
AH: Adenoid hypertrophy
CBCT: Cone beam computed tomography
DICOM: Digital imaging and communications
LU: Lumen
NE: Flexible fiberoptic nasal endoscopy
NPV: Negative predictive value
OHNS: Otolaryngology-head and neck surgery specialist
PPV: Positive predictive value
Se: Sensitivity
Sp: Specificity
UAW: Upper airway
SDB: Sleep-disordered breathing.
ICC: Intraclass correlation coefficient
CI: Confidence interval
LU 2D: Picture of the lumen
AD 2D: Picture of the adenoid mass
LU 3D: Prototype of the lumen
AD 3D: Prototype of the adenoid mass
