# Supplementary material for: Development, validation and application of a 3D printed model depicting adenoid hypertrophy in comparison to a Nasoendoscopy
Source: Head Face Med. 2020 Mar 9;16:5. doi: 10.1186/s13005-020-00216-4 (PMC7061480; doi:10.1186/s13005-020-00216-4)
Supplement: Supplementary file 2 — Additional file 2. Flowchart of the sampling reasoning and study design. [file 13005_2020_216_MOESM2_ESM.pdf]

SAMPLE REASONING

Identification

List received from previous study (n=40)

Excluded after records checking **(n=8)** ; Reasons: does not have CBCT (n=2), does not have ENT assessment (n=6);

Identification criteria: have a NE and CBCT

Patients records identified through UofA CBCT database **(n=32)**

Reliability

ICC (n=12)

3 patients from each grade was selected for ICC purpose.

Sample Selection

Excluded after Exclusion criteria were applied (n=17)

(1) scans in which the defined airway was not clear or fully contained in the image at a threshold airway sensitivity of 35 (n=8) Grade 1 = 3; Grade 2, n = 4; Grade 3,n=1

(2) Had adenoid surgery before (Grade, n=1)

(3) Not having a clearly description of the adenoid assessment through NE by the ENT, (n=3); Grade 1, n=2; Grade 3, n=1.

(4) Does not have the CBCT performed in the same day of the NE (n=5)

(Grade 1, n=3 (SD, 11-38 days); Grade 3, n=1 (2 years); Grade 4, n=1 (201 days))

Eligibility

Eligible sample for 3D and 2D printing (n=15)

(Grade 1, n=6; Grade 2, n=4; Grade 3, n=3; Grade 4, n=2)

Included

Sample included to mimic the population proportion (n=14)

(Grade 1 and Grade 2- non-enlarged, n=9; Grade 3 and Grade 4- enlarged, n=5)

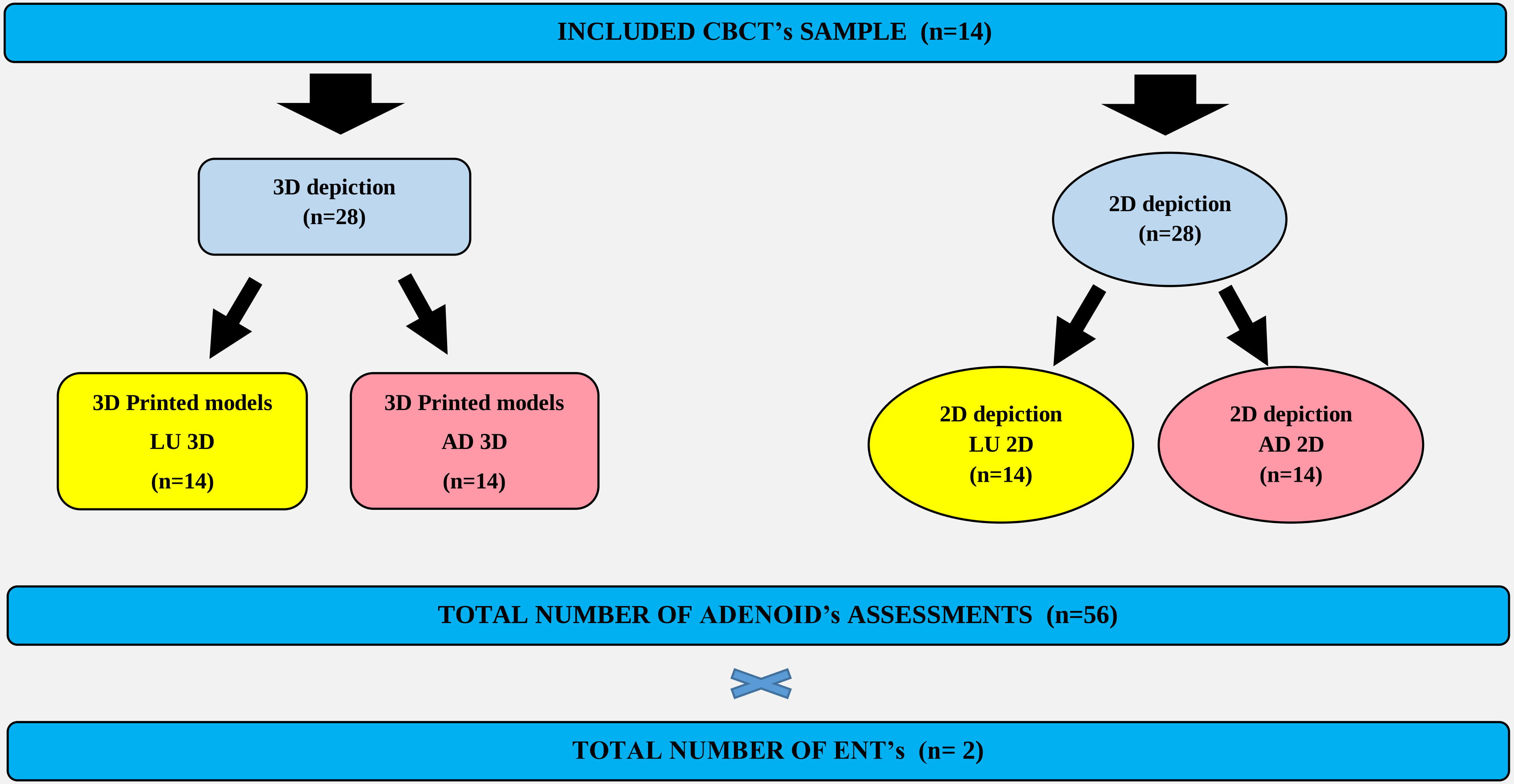
INCLUDED CBCT's SAMPLE (n=14)
3D depiction
(n=28)
2D depiction
(n=28)
3D Printed models
LU 3D
(n=14)
3D Printed models
AD 3D
(n=14)
2D depiction
LU 2D
(n=14)
2D depiction
AD 2D
(n=14)
TOTAL NUMBER OF ADENOID's ASSESSMENTS (n=56)
TOTAL NUMBER OF ENT's (n= 2)
