# Supplementary material for: Development, validation and application of a 3D printed model depicting adenoid hypertrophy in comparison to a Nasoendoscopy
Source: Head Face Med. 2020 Mar 9;16:5. doi: 10.1186/s13005-020-00216-4 (PMC7061480; doi:10.1186/s13005-020-00216-4)
Supplement: Supplementary file 1 — Additional file 1. Development of a 3D printed model of the nasopharyngeal adenoidal area using CBCT (Methodology). [file 13005_2020_216_MOESM1_ESM.docx]

Appendix A:

Development of a 3D printed model of the nasopharyngeal adenoidal area using CBCT (Methodology)

# INTRODUCTION

Partial or complete nasopharyngeal obstruction compromises an essential physiologic mechanism for humans to survive that is the breathing process. Breathing is also of significant importance to children’s craniofacial development and growth. Under normal conditions, one should breathe exclusively through the nose; when children cannot breathe enough air through the nose they start complementing their breathing intake by using the oral cavity. If this is chronic it may promote undesirable adaptations by the stomatognathic structures.[1-3] These breathing adaptations could alter deglutition, speech and mastication, ultimately leading to alterations in craniofacial growth and development.[4,5]

Back in 1661 adenoid hypertrophy (AH) was linked for the first time to speech problems, otitis, sleep apnea and cognitive problems.[6] Adenoids are a collection of lymphoepithelial tissues located near the oropharynx and nasopharynx. If adenoids become enlarged, they can interfere with airflow coming from the nasal structures to the lungs forcing children to also breathe through the mouth, which may lead to functional and/or anatomical adaptations.

AH is the most common cause of upper airway obstruction in children, with a prevalence ranging from 34% to 70% accordingly to a recent meta-analysis.[7] A recent systematic review suggested a close association between hypertrophic adenoids and upper airway obstruction.[8] Distinct studies[9,10,5,11] have linked AH to Sleep Disordered Breathing (SDB). SDB can have major negative effects in general health and quality of life. In addition, pediatric sleep disorders have been related to poor classroom performance.[12] Thus, in order to prevent craniofacial, overall health and behavioral alterations, an early AH diagnosis by a pediatric otolaryngology-head and neck surgery specialist (OHNS) is highly desirable.

Medical history (signs and symptoms) and physical examination combined to an objective exam is paramount for a precise upper airway obstruction diagnosis.[13,11,14,15] NE is the frequent chosen office procedure to assess sinus passage, nasal, and any nasopharyngeal related pathology by OHNS.[16,13,17,18] Studies by Chisholm et al. and Zalzal et al.[19,20] have cited Kubba et al., 2001 as first referring to NE as the “gold standard”. Nonetheless Kubba et al. 2001, did not state that; additionally, his study was performed under general anesthesia, which does not reflect the real scenario of a routine NE in most of the ENTs office.[21] NE under topic anesthesia, can be a distressing procedure for young children, NE could cause discomfort and pain to the patient, and needs cooperation in young children.[21,20,19,22] Besides the popularity of NE, radiographic images have been referred as better planning tools and are also important for diagnosis.[23] To date, there is no comprehensive guideline for assessing adenoidal enlargement in children.[13]

Lately attempts have been made to explore the use of CBCT imaging to assess the nasopharynx for screening purposes. CBCT imaging does not cause pain or discomfort, is less physically invasive, does not require sophisticated hand skills, and is usually more accepted by children when compared to NE. The main problem with using CBCT is the additional ionizing radiation exposure. CBCT has the potential to provide a 3D reconstruction of the nasopharynx and permit the assessment of potential HA obstruction. It is important to make it clear that the intention is not to request a CBCT for nasopharyngeal assessment but the fact that such craniofacial imaging could be readily available for other reasons and also be used for upper airway assessment.

Three-dimensional (3D) printing is a cutting-edge technology which has been increasingly used in medicine and dentistry.[24] The exploration of 3D printouts of the nasopharyngeal area has not been investigated yet. The goal of this chapter was to present reliability of the process to generate a 3D printed model of the nasopharyngeal area with distinct degrees of AH.

# MATERIAL AND METHODS:

The Research Ethics Committee at the University of Alberta approved the protocol number Pro00082445 for the present cross-sectional reliability study.

This study followed the ALARA principle (As Low As Reasonably Achievable) by the European Society of Radiology, thus, CBCTs were not specifically taken for this study. All imaging was pre-existing and were taken using the ICat scan (Classic, Cone Beam 3D Dental Imaging System; Imaging Sciences International, Hatfield, Pa). These were taken by using 6.19 mAs, at 110 kV, and with a customized height field of view maximum of 12 in, 0.3 mm voxel, and 8.9-second scan time with the patient in an upright position at maximum intercuspation. The DICOM data was analyzed using an automated reconstruction commercial software (Dolphin 3D, version 11.95 premium, Dolphin imaging & management Solutions, Chatsworth, CA, USA)

A list of 40 patients assessed at the Orthodontic Graduate Program and the Inter-disciplinary Airway Research Clinic at the University of Alberta. Inclusion criteria included having both a CBCT and NE assessment of the nasopharyngeal area done at the same day. Exclusion criteria included (1) scans in which the defined airway volume was not clear or fully contained in the image; (2) patients that had previous adenoid surgery, and (3) patients whose records did not have a clear description of the adenoid assessment through NE by the ENT, and (4) patients that did not have the CBCT performed on the same day as the NE. (Flowchart A)

For reliability purposes a sample of 12 CBCT - 3 subjects for each adenoid graded size[16] were randomly selected from the total sample. Two evaluators were previously calibrated using 4 randomly selected CBCT’s.

In order to standardize the assessment of the images a skull orientation was established as follows:

In the frontal view: (Figure 2.1A)

- **Axial plane**: determined at the level of anterior nasal spine
- **Mid-sagittal plane:** determined at the anterior nasal spine.

In the right view:

- Determined at the **coronal plane** oriented at the lateral orbital border at first. (Figure 2.1B)
- **Coronal slice (green line):** this second calibration was done right before the delimitation of the area of the interest. This second calibration was defined at the level of posterior nasal spine as seen in figure 2.1C.

The superimposition of the lower right and left mandible borders was checked, in the right view; as well as adjustments were made to rotate the volume clockwise, side-to-side, and up/down until the edges were as most coincident as possible. Then the left view was checked for any adjustment.

Bottom (facing up), and top (facing down) were also checked all at once for any additional orientation adjustment. Figure 2.1D shows the orientation plane in 3D view.

## Design and Fabrication

### Methodology for airway assessment (Lumen-LU) (Figure 2.2 to 2.4)

The delimitation of the nasopharyngeal area of interest was made on the sagittal and axial anatomical planes. We modified one previously developed method[25] used to measure the nasopharyngeal airway volume. The landmarks were selected based on the anatomical location of the adenoid gland, The Sinus/Airway tool in Dolphin software was used to select the landmarks as explained below:

- **Sagittal plane:** *sella turcica*, anterior tubercle of the atlas bone (C1), tip of the uvula and posterior nasal spine.
- **Axial plane:** posterior wall of maxillary sinus; right and left anterior point of cervical vertebrae, at the level of the most anterior point of the Atlas.

A seed point was placed in the airway space so that the software could capture the designated airway surface. The sensitivity tool from the semi-automated software was adjusted to 35, which was the value that better depicted the empty region (air) in our sample.

Figure 2.2 shows the landmarks in sagittal view (A) and axial view (B) as well as the seed point and the airway surface in yellow. Figure 2.3 shows a representation of the lumen (LU) surface in the translucent hard tissue view. Table 2.1 shows an example of the 3D LU volume surface representation (yellow) in bottom, top, front and right view for each one of the four different grades, depending on the level of adenoidal obstruction on the nasopharyngeal space. The grading ranges from 0 to almost complete obstruction. grade 1, when the obstruction is from 0-25%; grade 2, >25%-50%; grade 3, >50%-75%, and grade 4, > 75%.[16,26]

Table 2.2 shows the anatomical representation of the localization of region of interest (ROI) represented by the superimposition of the 3D LU surface within the hard tissue.

A schematic representation of the anatomical limits of the LU volume surface is shown in Figure 2.4.

### Methodology for AD assessment

For the adenoid assessment, the same orientation previously defined was followed, and the ROI was stablished on the following landmarks that was based on the modification of two previously used methods.[27,25]

**Sagittal** landmarks:

- Most inferior image of occipital bone;
- Most anterior point in spheno-occipital synchondrosis;
- Intersection point between palatal bone, and uvula anterior wall;
- Tip of uvula;

**Axial** landmarks were stablished at the level of Atlas:

- Most posterior wall of R and L maxillae (R and L paranasal sinus).
- two lateral R and L landmarks at the Atlas, at the level of the anterior tubercle

Two seed points were placed over the adenoid and the uvula, and the HU were set to -561 for the lower bound, and 3250 for the upper bound for all cases but one case in which the upper bound adjustment was not possible at 3250 and had to be set at 3052.

Delimitation of the region of interest (ROI) with the landmarks for the surrounding soft tissue (adenoid and soft palate) is shown in Figure 2.5. Table 2.3 shows the representation of the AD, and table 2.4 shows the anatomical representation of the localization of region of interest (ROI represented by the superimposition of the 3D AD volume surface within the hard tissue.

Figure 2.6 shows a schematic representation of the surrounding anatomical structures. Figure 2.7 shows the superimposition of the two models

## 3D printed model process

Both created volume surfaces that were saved into STL file and sent to the Centre for Teaching and Learning laboratory, at the University of Alberta.

The Afinia H800 3D printer (Chanhassen, MN, USA) (Figure 2.8) fabricated the models by uploading a file including the 3D image to its software, Afinia Studio software via USB to a HP laptop. Once uploaded on the software the size of the print was not changed to maintain the actual size of the anatomical structures. The printer used premium acrylonitrile-butadiene-styrene (ABS) filaments. The color of the filament was chosen as follows: yellow for the LU model, and red for the AD models. The filaments were loaded into the filament spool on the printer. For the adenoid models the printer used approximately 15 grams of ABS for each model; for the airway space model it used approximately 5.3 grams of ABS each.

The actual models created by the printer reproduced exactly the 3D surface generated by Dolphin software as seen in Figure 2.9. Figure 2.9 shows the 28 prototypes - representing grade1, grade2, grade 3 and grade 4.

## Statistical Analysis

The intra-class correlation coefficient (ICC) was used to assess intra and inter-reliability of both evaluators (CTB and LPP). Descriptive analysis of all data including means, standard deviation, minimum and maximum values were determined using the Statistical Package for the Social Science (IBM, version 25; SPSS Inc., Armonk, NY, USA). The level of significance and confidence interval (CI) was set at 0.05.

# RESULTS

As the calibration result, ICC was used to analyze the reliability. A high intra reliability was found for both types of models. For UA models an ICC =0.993 (95% CI:0.981-0.998) and for AD model an ICC =0.944 (95% CI: 0.861-0.982) in single measures, were found. Inter reliability was considered excellent between raters for both models; LU presented an ICC of 0.982; 95% CI, 0.939-0.995); and AD presented an ICC of 0.995; 95% CI, 0.981-0.998). Sample demographics and measurement Information can be seen in table 2.5

The volume measurements were assessed at three different times with three days washout period. A different spreadsheet was used between the measurements to secure blindness. The measurement error for LU was 1.667mm^3^ ± 1.000 mm^3^ (SD: 37.27 mm^3^), and for AD was 139.250mm^3^±1.006mm^3^ (SD: 98.46 mm^3^).

# DISCUSSION

This chapter refers to the second part of the present research project, which involves the design and development of the two 3D printed models. Reliability of the methodology was the key consideration in this design process. The intra-rater and the interrater reliability were confirmed by the ICC, and high reliability was found for both assessments.

The prototypes were developed using the DICOM data analyzed with commercial software Dolphin 3D. A previous study[28] had reconstructed an oropharynx airway with Mimics software, and a later study had compared both softwares, Dolphin and Mimics, and both showed less than 2% errors on volume measurement. We chose Dolphin software because it is considered the most commercial software used in maxillofacial surgery and in the orthodontics field in the north America, and the software has shown high reliability for airway measurement in previous studies.[28,29] Moreover, Dolphin software allows the created surface to be saved in an STL file, so we don’t need additional software for that purpose. Nonetheless, the same methodology could be applied to another software to verify its 3D printing feasibility.

Older versions of the software only allowed the measurement of the volume of empty areas[28], but the latest version of the software (11.95), the one we used on our study, allows for the measurement of the volume of tissues and bones due to an inverted threshold tool. As far as we know, our study is the first to report the technique of using the inverted tool, and to test the feasibility of the software in converting the surface to an STL file, as well as to generate a 3D printed prototype based on that.

In our study, we excluded cases in which CBCT and NE were not performed on the same day. The timing between the acquisition of CBCT DICOM and NE exam is critical since it is known that an increase in adenoid size can be correlated to an inflammatory and infectious episode[30], as well as with the level of immunological activity[31].

Density values, also known as gray thresholding, are still a challenge in the CBCT field, and to improve the airway research field, threshold values should be stated in every study. HU is not a standardized system, the HU value may not be the same if using the same CT machine but with a different technique, as well as if using a different machine.[32] Additional, it has been reported that the global thresholding pre-stablished in the automatic segmentation is the main cause of the largest variance compared to manual segmentation.[33,34] The use of different thresholds even for different anatomical sites has been suggested.[33] Therefore, the need for adjustment of the threshold has been explored to obtain less variation on the results.[28]

We conducted a literature search on airway studies using Dolphin software and we found six studies[35,25,29,28,36,34] that had reported adjustment of the threshold values. One study[28] obtained their scans through an I-CAT, and their scanning protocol was 120 kV, 5mA, 13x17cm field of view (FOV), 0.25mm voxel. In their study[28], they tested the sensitivity of 25, 50, and 70-75, and conclude that a threshold of 70 or over was the most accurate. In another study[35], the operator adjusted on a case-by-case basis, probably due to the anatomic abnormality of the craniosynostosis patient, since a previous study had stated that the gray values of air found in the nasal cavity differ from other areas due to the proximity of bones and mucous membranes.[33] In another study[25] two different thresholds (25 and 75) were used in two distinct samples where scans were obtained using different machines and scan protocols. Two studies[29,34] did not report the value of the adjustment. Table 2.6 shows the summary of the literature reviews and more specific information about the studies.

In the present study, we adjusted the threshold to a value of 35 HU because a threshold of 50 HU overlapped the surrounding structures, and a threshold of 25 HU did not fully fill the lumen in some cases. A previous study[25] at our institution used a threshold of 25 HU in a different sample of patients but under the same scanning protocol. We assume these threshold’s difference probably happened because those patients were wearing orthodontic apparatuses that can interfere with the CBCT phantom, consequently interfering at the gray thresholding.

Under similar conditions the selection of the cut off HU can have a major bearing in the volume measurement results as well as in the 3D surface. To simulate the impact of the changes we created a scenario with six threshold values (15, 25, 35, 50, 70 and 75 HU) in the same patient in the same landmark. These threshold values were based on previous airway studies[34,36,28,25,37] that reported the HU value. We observed results ranging from 4736 to 13163 in the surface volume, and in the sagittal area values ranging from 166 to 351, respectively from 15 to 75 HU. Additional, distortion of the 3D surface could be seen in threshold of 50 and over, as seen in Figure 2.10. Thus, caution should be taken since an inexperienced researcher would have unreliable results if any comparison in the measurements is made without standardizing the threshold sensitivity tool.

The 3D models were designed with a focus on the reproduction of actual nasopharyngeal anatomy, to demonstrate the lumen for airflow, but with some design adjustment, the same technique could be applied for different purposes. One example is the development of an algorithm, another would be that the use of the anatomical model for educational purpose for assessing the nasopharynx by adaptation of the technique and modification of the ROI making its use for basic scope navigation.

# LIMITATIONS

The HU selection is still a limitation in the upper airway CBCT field. More studies exploring the effect of different–related techniques are needed.

# CONCLUSION

The methodology for the generation of printed models of the nasopharynx’s lumen and of the adenoid and anatomical surrounding areas based on CBCT’s DICOM using Dolphin software is feasible and highly reliable.

### FUTURE RESEARCH

- Further, we would like to assess the educational applicability of the 2D and 3D, one nasopharyngeal space reconstructed in multiple planes and the other a 3D printed model of the same area both to simulate the likely view that OHNS would have through NE.
- With our AD prototype as a reference standard we would be able to access the most reliable CBCT threshold for our institutional studies on airways, based on the volume of water necessary to fill the empty spaces of the prototype measured with a high-precision micropipette.
- A matched sample with similar age, height, weight, and BMI would compensate the confounders and improve the analyses and correlation of the volume parametric measurements with the grading size;
- Development of an algorithm for adenoid hypertrophy screening in CBCT.

### Figures & Tables

Figure 2. 1- Plane orientation and calibration

Figure 2. 2- Delimitation of the nasopharyngeal region of interest. Sagittal (A) and Axial (B) as well as the seed point and the airway lumen in yellow color.

Figure 2. 3- Representation of the airway surface in the translucent hard tissue view. (A) sagittal view, (B) frontal view

Figure 2. 4- Airway surface schematic representation

Figure 2. 5- Sagittal (A) and axial (B) landmarks for adenoid and surrounded area delimitation

Figure 2. 6- Schematic representation of the adenoid mass surface

Figure 2. 7- Superimposition of the two 3D models

Figure 2. 8- Afinia H800 3D printer

Figure 2. 9-3D printed models (prototypes)

Figure 2. 10-Impact of sensitivity threshold adjustment in measurement and in 3D reconstruction surface in Dolphin software

Table 2. 1- 3D lumen surface (yellow color) in bottom, top, front and right view

Table 2. 2- 3D Lumen surface plus hard tissue representation

Table 2. 3-Adenoid mass 3D surface and grade size classification

Table 2. 4-3D Adenoid mass surface plus hard tissue representation

Table 2. 5- Demographics and measurements (mean) of the selected sample

Table 2. 6- Summary of the literature review in Dolphin software and interactive Imaging thresholding tool

## References

1. Aboudara C, Nielsen I, Huang JC, Maki K, Miller AJ, Hatcher D (2009) Comparison of airway space with conventional lateral headfilms and 3-dimensional reconstruction from cone-beam computed tomography. American Journal of Orthodontics & Dentofacial Orthopedics 135 (4):468-479. doi:<https://dx.doi.org/10.1016/j.ajodo.2007.04.043>

2. Abramson ZR, Susarla S, Tagoni JR, Kaban L (2010) Three-dimensional computed tomographic analysis of airway anatomy. Journal of Oral Maxillofacial Surgery 68 (2):363-371. doi:10.1016/j.joms.2009.09.086

3. Hatcher DC (2012) Cone beam computed tomography: craniofacial and airway analysis. Dental Clinics of North America 56 (2):343-357. doi:10.1016/j.cden.2012.02.002

4. Di Carlo G, Polimeni A, Melsen B, Cattaneo PM (2015) The relationship between upper airways and craniofacial morphology studied in 3D. A CBCT study. Orthod Craniofac Res 18 (1):1-11. doi:10.1111/ocr.12053

5. Guilleminault C, Akhtar F (2015) Pediatric sleep-disordered breathing: New evidence on its development. Sleep medicine reviews 24:46-56

6. Ruben RJ (2017) The adenoid: Its history and a cautionary tale. The Laryngoscope 127 Suppl 2:S13-s28. doi:10.1002/lary.26634

7. Pereira L, Monyror J, Almeida FT, Almeida FR, Guerra E, Flores-Mir C, Pacheco-Pereira C (2018) Prevalence of adenoid hypertrophy: A systematic review and meta-analysis. Sleep medicine reviews 38:101-112. doi:10.1016/j.smrv.2017.06.001

8. Becking BE, Verweij JP, Kalf-Scholte SM, Valkenburg C, Bakker EW, van Merkesteyn JP (2017) Impact of adenotonsillectomy on the dentofacial development of obstructed children: a systematic review and meta-analysis. European journal of orthodontics 39 (5):509-518. doi:10.1093/ejo/cjx005

9. Bozzini MF, Di Francesco RC (2016) Managing obstructive sleep apnoea in children: the role of craniofacial morphology. Clinics (Sao Paulo, Brazil) 71 (11):664-666. doi:10.6061/clinics/2016(11)08

10. Brambilla I, Pusateri A, Pagella F, Caimmi D, Caimmi S, Licari A, Barberi S, Castellazzi AM, Marseglia GL (2014) Adenoids in children: Advances in immunology, diagnosis, and surgery. Clinical Anatomy 27 (3):346-352. doi:10.1002/ca.22373

11. Kaditis AG, Alvarez MLA, Boudewyns A, Alexopoulos EI, Ersu R, Joosten K, Larramona H, Miano S, Narang I, Trang H (2016) Obstructive sleep disordered breathing in 2-to 18-year-old children: diagnosis and management. European Respiratory Journal 47 (1):69-94

12. Brockmann PE, Bertrand P, Castro-Rodriguez JA (2014) Influence of asthma on sleep disordered breathing in children: a systematic review. Sleep Medicine Review 18 (5):393-397. doi:10.1016/j.smrv.2014.01.005

13. Sharifkashani S, Dabirmoghaddam P, Kheirkhah M, Hosseinzadehnik R (2015) A new clinical scoring system for adenoid hypertrophy in children. Iranian journal of otorhinolaryngology 27 (78):55

14. Caylakli F, Hizal E, Yilmaz I, Yilmazer C (2009) Correlation between adenoid-nasopharynx ratio and endoscopic examination of adenoid hypertrophy: a blind, prospective clinical study. Int J Pediatr Otorhinolaryngol 73 (11):1532-1535. doi:10.1016/j.ijporl.2009.07.018

15. Major MP, Saltaji H, El-Hakim H, Witmans M, Major P, Flores-Mir C (2014) The accuracy of diagnostic tests for adenoid hypertrophy: a systematic review. Journal of the American Dental Association (1939) 145 (3):247-254. doi:10.14219/jada.2013.31

16. Parikh SR, Coronel M, Lee JJ, Brown SM (2006) Validation of a new grading system for endoscopic examination of adenoid hypertrophy. Otolaryngology—Head and Neck Surgery 135 (5):684-687

17. Feres MF, Hermann JS, Cappellette M, Jr., Pignatari SS (2011) Lateral X-ray view of the skull for the diagnosis of adenoid hypertrophy: a systematic review. Int J Pediatr Otorhinolaryngol 75 (1):1-11. doi:10.1016/j.ijporl.2010.11.002

18. Steen LH (2015) Design and validation of a practical simulator for the development of basic nasal endoscopy skills in otolaryngology – head and neck surgery. University of Alberta,

19. Chisholm E, Lew-Gor S, Hajioff D, Caulfield H (2005) Adenoid size assessment: a comparison of palpation, nasendoscopy and mirror examination. Clinical otolaryngology: official journal of ENT-UK; official journal of Netherlands Society for Oto-Rhino-Laryngology & Cervico-Facial Surgery 30 (1):39-41

20. Zalzal HG, Carr M, Kohler W, Coutras SW (2018) Adenoid Size by Drug Induced Sleep Endoscopy Compared to Nasopharyngeal Mirror Exam. International Journal of Pediatric Otorhinolaryngology

21. Kubba H, Bingham BJ (2001) Endoscopy in the assessment of children with nasal obstruction. The Journal of Laryngology & Otology 115 (5):380-384

22. Lertsburapa K, Schroeder Jr JW, Sullivan C (2010) Assessment of adenoid size: A comparison of lateral radiographic measurements, radiologist assessment, and nasal endoscopy. International journal of pediatric otorhinolaryngology 74 (11):1281-1285

23. Saedi B, Sadeghi M, Mojtahed M, Mahboubi H (2011) Diagnostic efficacy of different methods in the assessment of adenoid hypertrophy. American journal of otolaryngology 32 (2):147-151

24. Zaharia C, Gabor A-G, Gavrilovici A, Stan AT, Idorasi L, Sinescu C, Negruțiu M-L (2017) Digital Dentistry—3D Printing Applications. Journal of Interdisciplinary Medicine 2 (1):50-53

25. Thereza-Bussolaro CO, Hee So; Lagravère, M; Flores-Mir, C. (2019) Pharyngeal Dimensional changes in Class II malocclusion treatment when using Forsus® or intermaxillary elastics - An Exploratory Study. International Orthodontics 4th (December)

26. Ysunza A, Pamplona MC, Ortega JM, Prado H (2008) Video fluoroscopy for evaluating adenoid hypertrophy in children. Int J Pediatr Otorhinolaryngol 72 (8):1159-1165. doi:10.1016/j.ijporl.2008.03.022

27. Pacheco-Pereira C, Alsufyani NA, Major MP, Flores-Mir C (2016) Accuracy and reliability of oral maxillofacial radiologists when evaluating cone-beam computed tomography imaging for adenoid hypertrophy screening: a comparison with nasopharyngoscopy. Oral Surgery, Oral Medicine, Oral Pathology and Oral Radiology 121 (6). doi:<https://dx.doi.org/10.1016/j.oooo.2016.03.010>

28. Alves Jr M, Baratieri C, Mattos CT, Brunetto D, da Cunha Fontes R, Santos JRL, de Oliveira Ruellas AC (2012) Is the airway volume being correctly analyzed? American Journal of Orthodontics and Dentofacial Orthopedics 141 (5):657-661

29. Weissheimer A, de Menezes LM, Sameshima GT, Enciso R, Pham J, Grauer D (2012) Imaging software accuracy for 3-dimensional analysis of the upper airway. American Journal of Orthodontics and Dentofacial Orthopedics 142 (6):801-813

30. Licameli GR TD (2013) Pediatric otorhinolaryngology : diagnosis and treatment.

31. Modrzynski M, Zawisza E (2007) The influence of birch pollination on the adenoid size in children with intermittent allergic rhinitis. International journal of pediatric otorhinolaryngology 71 (7):1017-1023

32. Molteni R (2013) Prospects and challenges of rendering tissue density in Hounsfield units for cone beam computed tomography. Oral surgery, oral medicine, oral pathology and oral radiology 116 (1):105-119

33. Alsufyani NA, Noga ML, Witmans M, Major PW (2017) Upper airway imaging in sleep-disordered breathing: role of cone-beam computed tomography. Oral Radiology:1-9

34. Valerie R, Saridin JK, Bouw F, Murawska MM, Koudstaal MJ (2014) Measuring upper airway volume: accuracy and reliability of Dolphin 3D software compared to manual segmentation in craniosynostosis patients. Journal of Oral and Maxillofacial Surgery 72 (1):139-144

35. Pinheiro ML, Yatabe M, Ioshida M, Orlandi L, Dumast P, Trindade-Suedam IK (2018) Volumetric reconstruction and determination of minimum crosssectional area of the pharynx in patients with cleft lip and palate: comparison between two different softwares. Journal of Applied Oral Science 26:04. doi:<https://dx.doi.org/10.1590/1678-7757-2017-0282>

36. Guijarro-Martinez R, Swennen GR (2013) Three-dimensional cone beam computed tomography definition of the anatomical subregions of the upper airway: a validation study. Int J Oral Maxillofac Surg 42 (9):1140-1149. doi:10.1016/j.ijom.2013.03.007

37. Shi H, Scarfe WC, Farman AG (2006) Upper airway segmentation and dimensions estimation from cone-beam CT image datasets. International Journal of Computer Assisted Radiology and Surgery 1 (3):177-186
